# Evaluation of Clinical Alvarado Scoring System and CT Criteria in the Diagnosis of Acute Appendicitis

**DOI:** 10.1155/2016/9739385

**Published:** 2016-05-03

**Authors:** Idil Gunes Tatar, Kerim Bora Yilmaz, Alpaslan Sahin, Hasan Aydin, Melih Akinci, Baki Hekimoglu

**Affiliations:** ^1^Department of Radiology, Ankara Diskapi Training and Research Hospital, Ankara, Turkey; ^2^Department of General Surgery, Ankara Diskapi Training and Research Hospital, Ankara, Turkey

## Abstract

*Aim*. The aim was to evaluate the clinical Alvarado scoring system and computed tomography (CT) criteria for the diagnosis of acute appendicitis.* Material and Methods*. 117 patients with acute abdominal pain who underwent abdominal CT were enrolled in this retrospective study. Patient demographics, clinical Alvarado scoring, CT images, and pathologic results of the patients were evaluated.* Results*. 39 of the 53 patients who were operated on had pathologically proven acute appendicitis. CT criteria of appendiceal diameter, presence of periappendiceal inflammation, fluid, appendicolith, and white blood cell (WBC) count were significantly correlated with the inflammation of the appendix. The best cut-off value for appendiceal diameter was 6.5 mm. The correlation between appendiceal diameter and WBC count was 80% (*P* = 0.01 < 0.05). The correlation between appendiceal diameter and Alvarado score was 78.7% (*P* = 0.01 < 0.05).* Conclusion*. Presence of CT criteria of appendiceal diameter above 6.5 mm, periappendiceal inflammation, fluid, and appendicolith should prompt the diagnosis of acute appendicitis. Since patients with acute appendicitis may not always show the typical signs and symptoms, CT is a helpful imaging modality for patients with relatively low Alvarado score and leukocytosis and when physical examination is confusing.

## 1. Introduction

Acute appendicitis is the most common cause of acute surgical abdomen, with an estimated lifelong risk of 8.6% in men and 6.7% in women [1]. It is often regarded as a disease of the young population with a peak incidence in the second and third decades of life [1, 2]. Appendectomy is generally accepted as a first-line treatment for noncomplicated acute appendicitis. Reports have shown that preoperative radiographic evaluation has helped to decrease negative appendectomy rates from 20% to as low as 5% [3]. Computed tomography (CT) has been frequently used as an imaging modality in the evaluation of acute appendicitis and has improved the diagnostic ability leading to a significant reduction in the number of negative appendectomies [4]. With a reported sensitivity of up to 96.5% and specificity of about 98%, CT plays a major role in the clinical decision-making process in acute appendicitis and is considered as a first-line imaging modality in the diagnostic work-up for suspected acute appendicitis [5–8].

In 1986, Alvarado presented a clinical scoring system on the basis of eight predictive clinical factors to improve the accuracy of physicians' clinical assessments in diagnosing acute appendicitis. This scoring system produces a maximum total score of 10 points and includes clinical symptoms (nausea and anorexia), signs (fever, shifting pain, right lower quadrant pain, and rebound tenderness), and laboratory findings (leukocytosis and neutrophilia). Right lower quadrant pain and leukocytosis contribute 2 points each while the rest contributes 1 point [9].

The goal of the study was to analyze the CT criteria and clinical Alvarado scoring system and to find out the best cut-off value for appendiceal diameter in the diagnosis of acute appendicitis.

## 2. Materials and Methods

Following the approval of the institutional review board, the research was carried out retrospectively analyzing patient demographics, Alvarado clinical assessment scoring, and radiologic and pathologic results of the patients who had undergone abdominal CT for acute abdomen in our hospital.

### 2.1. Patients

A total of 117 patients who had abdominal CT for acute abdomen, within 24–48 hours after the beginning of the acute pain, were analyzed in this retrospective study (male, 83 (70.9%); female, 34 (29.1%); mean age 43 years; range, 16–78 years).

### 2.2. Imaging Technique

CT examinations were done on Philips MX8000 four-detector row scanner. The patients were scanned in supine position from the level of the liver dome to the symphysis pubis. 100–120 mL iodinated contrast medium was injected via the antecubital vein at a rate of 3 mL/second with a delay of 60 seconds between contrast administration and data acquisition. 5 mm thick axial images were obtained. Soft tissue kernel was used and reconstruction increment was 1 mm.

### 2.3. Image Interpretation

A single radiologist with 8 years of experience performed all measurements and interpreted the CT criteria except for the equivocal cases. In equivocal cases a decision was made with consensus of the same radiologist and another radiologist with 12 years of experience. Both readers were blind to the postoperative notes and pathology results. On 5 mm thick axial CT images they measured the appendiceal diameter and analyzed the presence or absence of inflammation, free fluid, and appendicolith. Pathological diagnosis was used as the reference standard.

CT evaluation of the appendix was based on four criteria: diameter of the appendix, periappendiceal inflammation, presence of extraluminal fluid collection around the appendix, and appendicolith [10].

- Diameter: the diameter of appendix was measured at the greatest portion of the visible appendix on axial scans. If the appendix was not seen the appendix was traced in coronal reformat images.
- Appendicolith: an appendicolith was defined as a well-defined high attenuation structure of any size within the appendix. Presence or absence of appendicolith was noted.
- Inflammation: the presence or absence of periappendiceal inflammation was analyzed. If it was present the degree of inflammation was categorized visually into two groups: mild to moderate and severe. If the periappendiceal fat stranding was present in up to one centimeter periphery of the appendix it was termed mild to moderate inflammation; if it included a larger area it was termed severe inflammation.
- Free fluid: presence or absence of free fluid which is suggestive of perforation and abscess formation was evaluated.

### 2.4. Statistical Analysis

All statistical analyses were performed using SPSS version 18.0 (SPSS Inc., Chicago, IL, USA). After the analysis of the patient files demographics, laboratory findings, clinical Alvarado scores, and CT interpretations were compared between patients with normal appendix and acute appendicitis.

Pearson's chi-square test was used to analyze the relation of the sex, Mann-Whitney *U* test for the relation of age, appendiceal diameter, and white blood cell in patients with normal appendix and acute appendicitis. Pearson's chi-square test was used to analyze the correlation between periappendiceal inflammation, fluid, appendicolith, and inflammation of the appendix. Spearman's Correlation Coefficient was utilized to evaluate the correlation between appendiceal diameter and WBC.

## 3. Results

In the retrospective analysis of the patient files 53 patients were taken to appendectomy surgery (male, 37 (69.8%); female, 16 (30%); mean age, 43 years; range, 16–72 years). Thirty-nine of them had pathologically proven acute appendicitis (male, 28 (71.8%); female, 11 (28.2%); mean age, 41 years; range, 16–72 years). The remaining 14 patients had clinical acute appendicitis judged by the surgeon but the histopathology of the patients was normal. The negative appendectomy rate was %26.41. According to the pathological results there were 5 false negative (9.4%) and 2 false positive (3.7%) CT interpretations.

Sixty-four patients with abdominal pain had nonsurgical treatment. According to the follow-up of these patients 7 patients were diagnosed with nephrolithiasis, 9 with ureterolithiasis, 2 with pyelonephritis, 5 with cholecystitis, 2 with pancreatitis, 8 with subileus, 14 with diverticulitis, 2 with ulcerative colitis, 3 with Crohn's disease, 5 with mesenteric lymphadenitis, 1 with epiploic appendicitis, 2 with ovarian cyst, 1 with pelvic inflammatory disease, and 3 with familial Mediterranean fever.

When the patients with pathologically proven acute appendicitis were analyzed, sex was not related to the inflammation of the appendix (*P* = 0.886 > 0.05). Age of the patients was also not related to the inflammation of the appendix (*P* = 0.669 > 0.05).

Appendiceal diameter and white blood cell (WBC) were correlated to the inflammation of the appendix (*P* = 0.001 < 0.05). The patients with acute appendicitis had a mean appendiceal diameter of 8.5 mm (range, 6–16; SD, 2.7) and a mean WBC count of 14.4 × 10^9^/L (range, 5.8–30.3; SD, 5) whereas the patients with normal appendix had a mean appendix diameter of 3.1 mm (range, 2–5; SD, 0.8) and a mean WBC count of 6.6 × 10^9^/L (range, 3.5–13; SD, 1.6). The mean Alvarado score of the patients with acute appendicitis was 6.6 (range, 4–10; SD, 1.7).

**Figure 1:**
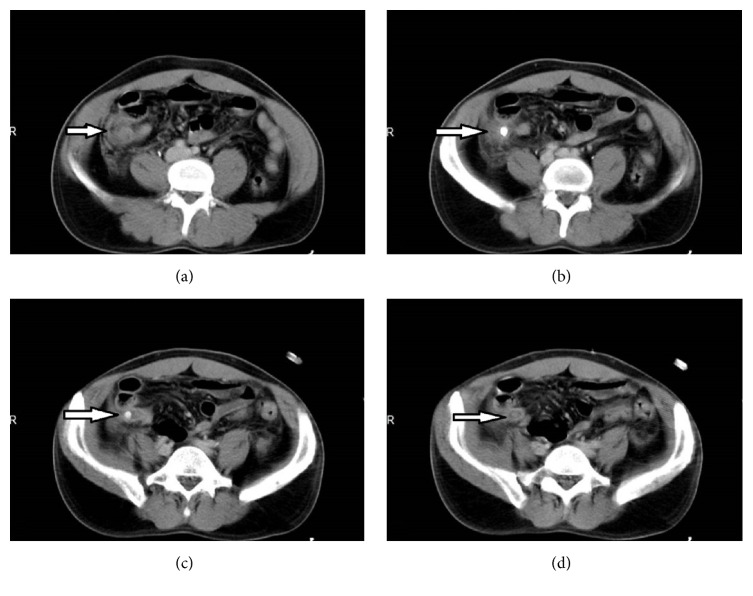
IV contrast enhanced axial abdominal CT images of a 43-year-old man with positive appendectomy. (a) Periappendiceal fat stranding and free fluid, (b) appendicolith in appendix lumen and periappendiceal free fluid, (c) appendicolith in dilated appendix lumen measuring 8 mm and periappendiceal fat stranding, and (d) wall thickening of dilated appendix.

There was a correlation between the CT criteria of presence of periappendiceal inflammation, fluid, appendicolith, and inflammation of the appendix (*P* = 0.01 < 0.05). In the patients with normal appendix mild to moderate periappendiceal inflammation was noted in 10 patients (12.8%) and severe periappendiceal inflammation was present in 3 patients (3.8%). In the patients with acute appendicitis mild to moderate periappendiceal inflammation was observed in 12 patients (30.8%) and severe periappendiceal inflammation was seen in 19 patients (48.7%). None of the patients with normal appendix had appendicolith whereas 13 patients (33.3%) with acute appendicitis demonstrated appendicolith. Nine patients (11.5%) with normal appendix presented with periappendiceal fluid; on the other hand 15 patients (38.5%) with acute appendicitis had periappendiceal fluid (Figure 1). The distribution of CT signs in patients with normal appendix and acute appendicitis is summarized in Table 1.

The best cut-off value for appendiceal diameter was found to be 6.5 mm with very high class prediction. The correlation between appendiceal diameter and WBC was 80% (*P* = 0.01 < 0.05). The correlation between appendiceal diameter and Alvarado score was 78.7% (*P* = 0.01 < 0.05).

## 4. Discussion

This study analyzed the CT criteria and clinical Alvarado scoring system in order to find out the best cut-off value for appendiceal diameter in the diagnosis of acute appendicitis. CT diagnosis of acute appendicitis can be based on four criteria which are appendiceal diameter, presence of appendicolith, periappendiceal inflammation, and free fluid. It is crucial to determine the maximum diameter of appendix with CT for accurate diagnosis of the acute appendicitis and to eliminate other etiologies of acute abdominal pain [11]. The inflamed appendix is distended with a diameter measuring between 6 and 40 mm and a wall thickness of 1–3 mm [12]. The wall is usually asymmetrically thickened and is enhanced with intravenous contrast medium [13]. In this research the best cut-off value for appendiceal diameter was found to be 6.5 mm and there was a significant correlation between appendiceal diameter and Alvarado score.

Appendicoliths detected on CT are reported to be associated with severe appendicitis, appendiceal perforation, recurrent appendicitis after conservative therapy, or failure of antibiotic therapy [14]. Ishiyama et al. showed a significant relationship between the presence of appendicolith and the severity of acute appendicitis in a retrospective study with a total of 254 patients who had pathologically proved acute appendicitis. In multivariate analysis, they showed that the presence of appendicoliths and the location of an appendicolith at the root of the appendix were significantly associated with gangrenous appendicitis [15]. The authors suggest that it is probable that the root of the appendix may be easily obstructed by an appendicolith as the root of the appendix has a narrower lumen in comparison to the rest of the appendix. They further assert that an appendicolith can lead to severe disease, especially when it is a larger one or it is at the root of the appendix.

There are some specific surgeon alerting CT findings for perforation which is a complication of appendicitis. These are abscess, phlegmon, extraluminal air, extraluminal appendicolith, and focal defect in the enhanced wall of the appendix.

At CT, ascending retrocecal appendicitis has been reported to be associated with a high incidence of retroperitoneal inflammatory changes and appendiceal perforation. Periappendiceal inflammatory changes are most commonly observed in the retrocolic space, followed by paracolic gutter, pararenal space, mesentery, perirenal space, and subhepatic space. Perforation of the appendix with the formation of an abscess is present in approximately half of the cases [16].

The utility of Alvarado scoring system was widely researched in the literature. In a review of 233 patients with right lower quadrant pain, Pouget-Baudry et al. established that Alvarado scoring was very useful. Authors found out that score 6 was correlated well with the presence of appendicitis and score 4 was correlated well with the absence of appendicitis. They suggested that observation or complementary tests (i.e., ultrasound or CT) should be used only in the case of a score between 4 and 6 [17]. McKay and Shepherd recommended surgical consultation if clinical presentation suggested acute appendicitis by an Alvarado score of 7 or higher. They reported that computed tomography was not indicated in patients with Alvarado scores of 3 or lower to diagnose acute appendicitis [18].

Wang et al. researched the use of CT in patients with suspected acute appendicitis who had relatively low Alvarado scores [19]. Sixty patients with suspected acute appendicitis and an Alvarado score between 4 and 7 points were considered in a prospective study. Clinical and laboratory differences were compared between patients with histologically proven acute appendicitis and patients without acute appendicitis. Authors evaluated whether the use of CT could be decreased in patients who were less likely to have acute appendicitis. They concluded that CT is necessary for patients with relatively low Alvarado score when leukocytosis is noted.

Nelson et al. carried out a retrospective study to examine the relevance of clinical assessment in diagnosing appendicitis in the era of routine use of CT in a total of 664 patients. In cases of high clinical suspicion (i.e., in cases with Alvarado score of 7) the surgeon's clinical assessment was reliable whereas, in cases in which the surgeon's initial impression was low for acute appendicitis, 87% of these patients had confirmed appendicitis on final pathology. Their results suggested that the surgeon's overall clinical assessment was imperfect at best. The authors concluded that although physical examination remains crucial, CT has become the primary modality dictating care of patients with presumed appendicitis [20].

Patients with acute appendicitis do not always show the typical signs and symptoms. The clinical features in children are usually atypical, with generalized abdominal pain in contrast to typical localized pain. The diagnosis of acute appendicitis might also be delayed in the elderly since there is a wide range of differential diagnosis due to the increased incidence of age-related diseases such as diverticulitis.

Elderly patients may account for nearly 10% of cases referred for CT for suspected appendicitis [21]. The classic presentation of appendicitis involving the triad of fever, leukocytosis, and right lower quadrant pain is present in only 10–26% of patients over 60 years of age [22]. Treating elderly patients may pose a challenge since treatment modality in the majority of cases of acute appendicitis is surgery. Given that the elderly patients are more prone to have relevant comorbidities, the elderly are at increased risk for complications related to both delayed diagnosis of appendicitis and unnecessary appendectomy. The overall mortality rate for elderly patients with appendicitis has been published to be about 15% [23]. An accurate diagnostic test for acute appendicitis is therefore very crucial in elderly patients with suspected appendicitis.

An accurate diagnosis of acute abdomen is important in distinguishing surgical conditions like acute appendicitis from nonsurgical conditions that may have a similar presentation. Various pathologies might mimic appendicitis on CT imaging. These include right-sided diverticulitis, cecal carcinoma, Crohn's colitis, mesenteric inflammation, complicated ovarian cysts, endometriosis, ectopic pregnancy, local lymphadenopathy, and fibrofatty proliferation [24]. In a patient with a normal appendix the setting of acute abdomen may be related to tuboovarian abscess, epiploic appendicitis, biliary colic, or urinary tract infection. Perforated duodenal ulcer, superior mesenteric venous thrombosis, small bowel ischemia, and abdominal wall hernia are conditions which present with right lower abdominal pain and are treated surgically [25]. A surgeon's clinical evaluation alone can reliably diagnose acute appendicitis in highly suspicious cases of appendicitis without the help of CT. Nevertheless the surgeon's assessment may miss the cases in patients meeting few diagnostic criteria. It is these patients in whom CT becomes an effective and useful adjunct in the workup of acute appendicitis. In the literature the rate of negative appendectomy has been reported as high as 17–36% without the use of CT [26]. The negative appendectomy rate in our study was 26.41% which was relatively high that needs to be lowered with further research and quality monitoring.

Radiologic diagnosis of acute appendicitis can be missed, especially when the patients have equivocal CT findings [27]. Appendicitis is present in up to 30% of patients with equivocal CT findings [28]. As a result, in spite of the progress in CT techniques, negative appendectomy and delayed diagnosis may still occur.

A recent meta-analysis of CT use in the evaluation of suspected acute appendicitis suggested that routine CT in all patients presented with suspected appendicitis could reduce the rate of unnecessary surgery without increasing morbidity [29]. In the diagnosis of suspected acute appendicitis, CT has been reported to decrease the incidence of negative appendectomy [30].

The role of ultrasonography should also be emphasized in the diagnosis of acute appendicitis since it is a widely available, affordable modality which does not utilize ionizing radiation. It has been reported to have a sensitivity between 55 and 98% and specificity of 78–100% in the literature. The limitations of this technique are the user dependancy and the difficulty to obtain good image quality in some patients [9, 31, 32].

## 5. Conclusion

CT is an accurate imaging modality for the diagnosis of acute appendicitis. Presence of CT criteria of appendiceal diameter above 6.5 mm, periappendiceal inflammation, fluid, and appendicolith should prompt the diagnosis of acute appendicitis. Even though the optimal use of CT in evaluating patients with suspected appendicitis is not clear, it is necessary for patients with relatively low Alvarado score and leukocytosis and also when physical examination is confusing.

## Figures and Tables

**Table 1:** The distribution of CT signs in patients with normal appendix and acute appendicitis.

Feature	Patients with normal appendix (*n* = 78)	Patients with acute appendicitis (*n* = 39)
(i) Appendiceal diameter	3.1 ± 0.8 mm, range: 2–5	8.5 ± 2.7 mm, range: 6–16
(ii) Mild-moderate inflammation	10/78 (12.8%)	12/39 (30.8%)
(iii) Severe inflammation	3/78 (3.8%)	19/39 (48.7%)
(iv) Free fluid	9/78 (11.5%)	15/39 (38.5%)
(v) Appendicolith	0/78 (0.0%)	13/39 (33.3%)

## Additional Points

Having been designed retrospectively, a limitation of this study is that it lacks the follow-up of the nonsurgically treated patients. The data suggest that a CT scan reliably identifies the patients in need of an operation for appendicitis and it seems to be as good as clinical examination. In this retrospective design the Alvarado score of the patients who did not undergo appendectomy was not present since it could be only calculated for the patients who were clinically suspected of acute appendicitis by the surgeons, because most of the patients who were not diagnosed with acute appendicitis were discharged from the hospital and sometimes not followed up in the same hospital. In a study designed prospectively the Alvarado scores can be evaluated in all patients with abdominal pain including the patients who are not clinically suspected of acute appendicitis. The low number of females included in the study is explained by the admission of most female patients with abdominal pain directly to hospitals specialized in women's health. There were no patients under the age of 16 since the study was conducted in an adult's hospital. Our results should be further validated with higher number of patients to build more solid recommendations.
